# Pursuing Investigations Establish the Challenging Diagnosis of Primary Aldosteronism

**DOI:** 10.7759/cureus.19613

**Published:** 2021-11-15

**Authors:** Than Aung, Haresh Tulsidas

**Affiliations:** 1 Internal Medicine, Singapore General Hospital, Singapore, SGP

**Keywords:** hypokalemia, secondary hypertension, adrenal adenoma, primary aldosteronism, aldosterone renin ratio

## Abstract

Primary aldosteronism is one of the causes of secondary hypertension. The most sensitive screening test for primary aldosteronism is the measurement of the plasma aldosterone concentration and plasma renin activity to calculate the aldosterone/renin ratio. We report a case of hypertension and hypokalemia with a negative plasma aldosterone/renin ratio, inconclusive confirmatory saline infusion test. Subsequently, the patient was diagnosed with primary aldosteronism secondary to adrenal adenoma. If there is a high index of clinical suspicion of primary aldosteronism, it is important to evaluate further to establish the diagnosis for initiation of specific treatment because failure to identify primary aldosteronism can lead to aldosterone-specific adverse cardiovascular diseases and events.

## Introduction

Primary aldosteronism is a frequently under-recognized cause of secondary hypertension. It is a condition caused by excessive secretion of aldosterone by adrenal glands. The two major causes of primary aldosteronism are aldosterone-producing adenoma and bilateral adrenal hyperplasia. The distinctive clinical presentation includes hypertension and hypokalemia. Diagnosis consists of the measurement of plasma aldosterone levels and plasma renin activity as an initial screening test. Treatment is different depending on the underlying etiology of primary aldosteronism either surgery or medical therapy. Excess aldosterone level is responsible for an increased risk of cardiovascular events. The appropriate treatment of primary aldosteronism can revere adverse cardiovascular effects. We report a case of a 50-year-old Chinese man who presented with hypokalemia and hypertension, unremarkable aldosterone/renin ratio, and indeterminate saline infusion test was eventually diagnosed to have primary aldosteronism.

## Case presentation

A 50-year-old Chinese man presented to our clinic for cough, productive sputum, and sore throat for a week. He also complained of a few months' history of passing frothy urine, and polyuria, which appeared worse last week. He had a past medical history of chronic kidney disease (CKD) stage 3 with proteinuria, type 2 diabetes mellitus, hypertension, and hyperlipidemia. He lost follow-up with his private physician and came to our clinic. His medications were irbesartan 300 mg daily, long-acting nifedipine 30 mg twice daily, spironolactone 25 mg daily, metformin 250 mg twice daily, and rosuvastatin 10 mg daily. He had a family history of coronary artery disease and diabetes mellitus in his father. He did not take herbal or traditional supplements. On physical examination, he appeared non-cushingoid. His blood pressure was 135/83 mmHg. There was no facial plethora, central obesity, or wasting of the proximal muscle of extremities suggestive of the presence of hypercortisolism. Apart from having mild bilateral ankle edema, the rest of the examination was normal. He was treated symptomatically as an upper respiratory tract infection and investigated further for polyuria and frothy urine. The laboratory results were shown in Table 1. 

**Table 1:** Laboratory findings eGFR CKD-EPI: estimated glomerular filtration rate (chronic kidney disease epidemiology collaboration); GFR: glomerular filtration rate; KDIGO: kidney disease improving global outcomes; ADA: American Diabetes Association

	Patient value	Reference range
Serum urea (mmol/L)	6.1	2.7-6.9
Serum sodium (mmol/L)	142	136-146
Serum potassium (mmol/L)	3.0	3.6-5.0
Serum chloride (mmol/L)	102	100-107
Serum bicarbonate (mmol/L)	29.5	19.0-29.0
Serum creatinine (umol/L)	138	54-101
eGFR (CKD-EPI) (mL/min/1.73m^2^)	51	Stage 1 GFR >90; Stage 2 GFR 60-89; Stage 3 GFR 30-59; Stage 4 GFR 15-29; Stage 5 GFR <15 by KDIGO classification
Urine protein creatinine ratio (g/g)	2.28	Normal range <0.2
Hemoglobin A1c (%)	6.8	6.5 or higher (diagnosis of diabetes mellitus by ADA criteria)

The serum creatinine was the baseline value for him. His CKD was stable last nine months with an estimated glomerular filtration rate (eGFR) of 51 mL/min/1.73m^2^ by chronic kidney disease epidemiology collaboration (CKD-EPI) creatinine formula. The value of urine protein creatinine ratio was similar to his precedent reading. He reported the levels of potassium in his blood results were often low, ranging from 3.0 to 3.3 mmol/L. Hypokalemia was evaluated by further spot urine potassium and spot urine creatinine. The results were as follows: urine creatinine 2551 umol/L, urine potassium 11 mmol/L, and urine potassium creatinine ratio 4.31 mmol/L which indicated that hypokalemia was due to increased renal loss of potassium [1]. Urine potassium value may depend on the amount of potassium in diet and the amount of potassium in the body. The urine creatinine can fluctuate based on diet, exercise, and hydration status. Concurrent measurement of urine potassium and urine creatinine can give a more accurate potassium excretion rate.

He took spironolactone for three years and irbesartan for many years to control his blood pressure. Both medications were discontinued to check for plasma aldosterone and renin. To control his blood pressure, he was started on hydralazine 25 mg three times a day, potassium 16 mmol four times a day, and the dose of nifedipine was increased to 60 mg in the morning and 30 mg at night. Doppler ultrasonography of renal arteries showed no evidence of renal artery stenosis. There were mildly raised renal echoes due to mild chronic renal parenchymal disease. After stopping spironolactone for four weeks, correction of hypokalemia with oral potassium supplements, and no dietary restriction of salt intake, biochemical evaluation for primary hyperaldosteronism was done in the morning with an upright position which showed plasma aldosterone concentration 15.0 ng/dL (21 ng/dL or less), plasma renin activity 4.8 ng/mL/h (2.9-10.8 ng/mL/h) and a calculated ratio of plasma aldosterone renin 3.12. Blood pressure readings were generally maintained below 140/90 mmHg. Considering the aldosterone/renin ratio was negative with hypokalemic hypertension, we decided to check the 8 a.m. serum cortisol level and repeat the aldosterone and renin level in the morning. Repeat aldosterone renin levels four weeks later together with morning serum cortisol showed that aldosterone 15.16 ng/dL, plasma renin activity 7.05 ng/mL/h, with calculated aldosterone/renin ratio 2.15 and serum cortisol 372 nmol/L (126-626 nmol/L).

Due to high suspicion of hyperaldosteronism and considering the possibility of false-negative aldosterone/renin ratio, a confirmatory and more specific test was conducted. Hypokalemia was corrected with a continued potassium supplement. His blood pressure and CKD were stable. The 24-hour urine potassium measurement was not performed because testing while on potassium supplement may affect the accuracy of the result. The salt loading test came out with an equivocal result. Plasma aldosterone concertation was 15.0 ng/dL before infusion and 8.7 ng/dL four hours after infusion. Diagnosis of primary aldosteronism is considered indeterminate when post-infusion plasma aldosterone level falls between 5 and 10 ng/dL [2].

**Figure 1:**
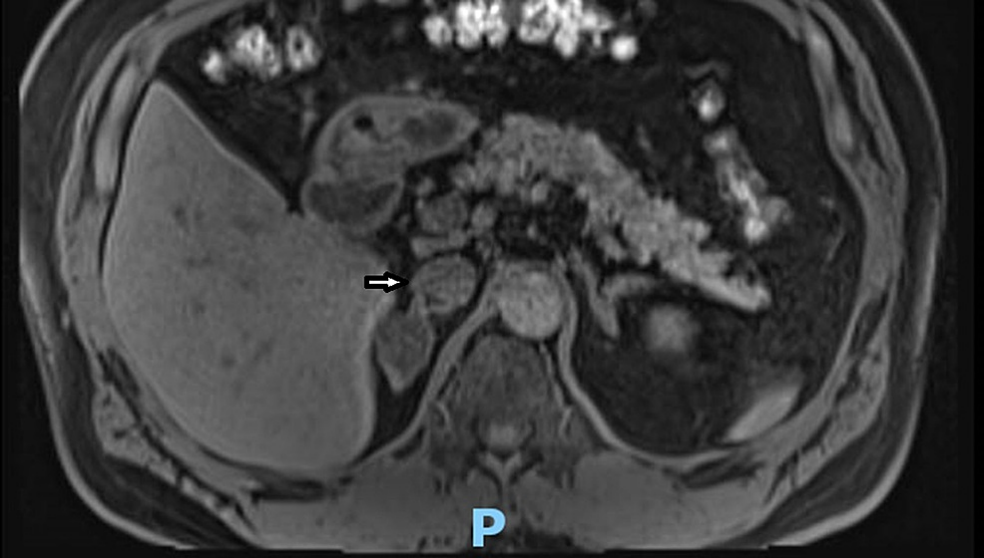
MRI adrenal glands There is a 2.4 x 2.0 cm ovoid nodule arising from the right adrenal gland lateral limb (pointed by an arrow). The left adrenal gland appears normal.

Cushing syndrome may be a possible differential diagnosis although he had no clinical features suggestive of hypercortisolism and the morning serum cortisol was normal. The patient remained on a high dose of potassium supplement. Although the aldosterone/renin ratio and saline infusion test results were not confirmed for primary aldosteronism, his unexplained hypokalemia and hypertension raised the index of suspicion for primary aldosteronism. Considering his CKD stage 3 and concern over the risk of contrast-induced nephropathy, magnetic resonance imaging (MRI) of the adrenal gland was performed consequently to look for structural adrenal abnormality. It reported a right adrenal nodule of 2.4 cm in size consistent with adrenal adenoma. The left adrenal gland appears normal (Figure 1).

Hypertension with long-standing hypokalemia, negative aldosterone/renin ratio, inconclusive salt infusion with a right adrenal nodule on imaging altogether indicated that he was likely to have primary aldosteronism due to adrenal adenoma. Two treatment options were offered: first, medical treatment with spironolactone, and second, removal of the right adrenal nodule by surgery. The patient expressed that he preferred to undergo surgical adrenalectomy. Spironolactone 50 mg daily was started. He continued on the same dosage of hydralazine, nifedipine, and potassium supplement, maintaining blood pressure optimally controlled. The patient was selected for surgery after imaging; there was limited time to titrate up the dose of spironolactone to achieve the least possible dose of potassium supplementation.

Measuring aldosterone level by adrenal vein sampling procedure was not performed because of his CKD stage 3. He had right adrenalectomy after a few months later. Morning plasma aldosterone and cortisol levels were checked on postoperative day one. Plasma aldosterone level was less than 4 ng/dL and cortisol level was 440 nmol/L. The patient had no clinical evidence of postoperative adrenal insufficiency. Potassium replacement and spironolactone were discontinued immediately after surgery. Histology reported a circumscribed nodule with incomplete compressed fibrous capsule, composed of trabeculae and nests of polygonal cells with clear to eosinophilic vacuolated cytoplasm and rounded nuclei, consistent with adrenal adenoma. After the operation, his potassium levels were in an upward trend from 3.7 to 4.1 mmol/L and he achieved a greater blood pressure lowering effect with nifedipine monotherapy. On subsequent visits, he maintained normal potassium levels and optimal control of blood pressure.

## Discussion

Primary hyperaldosteronism is an under-reported cause of hypertension. Studies reported primary aldosteronism may account for 5% to 10% of hypertensive patients [2]. The classic clinical indications suggestive of primary aldosteronism are hypertension and hypokalemia. However, only an estimated 9-37% with primary aldosteronism are hypokalemic and the recent studies of primary aldosteronism have reported that most of the patients have normokalemic hypertension at detection [2]. Patients with primary aldosteronism are associated with an increased risk of cardiovascular morbidity and mortality when compared to patients with essential hypertension. It is important to identify primary aldosteronism because early initiation of specific therapy results in improvement of hypertension and resolution of increased cardiovascular risk. Measuring plasma aldosterone/renin ratio is a commonly used guideline-recommended case-detection test in hypertensive patients suspicious for primary aldosteronism [2].

The accuracy of plasma aldosterone/renin ratio is highly variable across the studies and it has a sensitivity ranging from 10% to 100% and a good specificity from 70% to 100% reflecting that negative aldosterone/renin ratio cannot exclude the possibility of primary aldosteronism when screening aldosterone/renin ratio falls below traditionally established thresholds [3]. A plasma aldosterone/renin ratio greater than 30 is considered a positive screening test for diagnosis of primary aldosteronism, described in Endocrine Society guidelines [2]. There are higher sensitivity and specificity of 90% when the combination of a plasma aldosterone concentration equal to or above 20 ng/dL and a plasma aldosterone/renin ratio above 30 is applied in the primary detection test for the diagnosis of primary aldosteronism [4]. In our case, plasma aldosterone/renin ratio results were far below the recommended value to suspect the primary aldosteronism. Measuring plasma aldosterone/renin ratio as a detection test is simple and widely available, but the interpretation of a single morning sample result may not represent daily total aldosterone secretion.

The screening aldosterone and renin tests of our patient were performed in the morning in an upright position. A guideline-recommended threshold value of aldosterone/renin ratio may reflect the result of fluctuation of circulating levels of aldosterone throughout the day in patients with primary aldosteronism [5]. The plasma aldosterone/renin ratio results can be false-positive and negative because the diagnostic reliability of the test is affected by many factors. Hence, confirmatory testing for primary aldosteronism is recommended by the Endocrine Society. A saline infusion test was performed to confirm or exclude the diagnosis. Plasma aldosterone level is measured at the conclusion of an intravenous infusion of 0.9% saline. Diagnosis of primary aldosteronism is unlikely if post-infusion plasma aldosterone levels are less than 5 ng/dL, but aldosterone levels more than 10 ng/dL can confirm the diagnosis. Indeterminate diagnosis can be considered when aldosterone level falls between 5 and 10 ng/dL. The saline infusion test demonstrated a sensitivity of 81% and a specificity of 96% [2]. 

A study reported that the best balance between sensitivity and specificity could be achieved if a threshold value was set at 8 ng/dL for a confirmation test, with resultant a positive predictive value of 94% and a negative predictive value of 82% [6]. Our patient post-infusion aldosterone level was 8.7 ng/dL which fell in an inconclusive range based on Endocrine Society guidelines. However, the level was more than the cutoff value of the study mentioned above, we observed there was a high probability that he had primary aldosteronism. Guideline recommended adrenal imaging for patients with all primary aldosteronism to exclude adrenal masses which may represent adrenocortical carcinoma [2]. n consideration of persistent hypokalemia with hypertension, we led our patient to undergo adrenal imaging to explore the presence of an adrenal structural abnormality despite being inconclusive biochemical tests for the diagnosis of primary aldosteronism. Our patient received an MRI of adrenal glands as he expressed concern about the risk of contrast-induced nephropathy. MRI reported a right adrenal adenoma. The Endocrine Society guided that unilateral disease can be managed by either medical therapy with spironolactone or laparoscopic adrenalectomy.

Most of the patients retain better blood pressure control and normalize serum potassium concentrations after adrenalectomy. Half of the patients with aldosterone-producing adenoma achieved a cure for hypertension after operation [2]. Studies have shown marked improvements in adverse left ventricular changes including a left ventricular mass index at long term after surgery [7]. Adrenal vein sampling is a standard test and recommended to differentiate unilateral adenoma from bilateral hyperplasia when the patient is keen and suitable for the surgical option. However, measuring aldosterone level by adrenal vein sampling procedure was not performed in view of his chronic renal impairment.

Cushing syndrome may be a possible differential diagnosis in patients with hypokalemic hypertension and diabetes. In Cushing syndrome due to adrenal adenoma, there would be prolonged suppression of the hypothalamic-pituitary-adrenal axis and inhibition of adrenocorticotropic hormone (ACTH) secretion by cortisol excess with contralateral adrenal gland atrophy. The patient will require glucocorticoid replacement perioperatively to avoid the risk of Addisonian crisis after resection of adrenal adenoma because the recovery of ACTH secretion can be delayed. Our patient had no clinical evidence of acute steroid withdrawal postoperatively. In addition, his baseline 8 a.m. cortisol level and post-surgery cortisol level both were normal, 372 nmol/L and 440 nmol/L, respectively. All the above findings ruled out the possibility of Cushing syndrome secondary to adrenal adenoma. Renal tubular acidosis (RTA) can be considered as one of the causes of hypokalemia but bicarbonate will be low in RTA which is not applicable in our case. Renal vascular hypertension was also excluded by normal doppler ultrasonography of renal arteries. There is no family history of Liddle syndrome or syndrome of apparent mineralocorticoid excess and these conditions usually present in childhood. Our patient did not consume herbal or traditional supplements (e.g., licorice).

The findings of a significant fall in plasma aldosterone level, normalization of serum potassium, and better improvement in blood pressure with a single anti-hypertensive therapy after adrenalectomy demonstrated that he had primary aldosteronism secondary to adrenal adenoma. There was no hypokalemia and the potassium supplement was not required anymore.

The patients presenting with primary aldosteronism displayed an increased cardiovascular event than did the primary hypertension patients. The appropriate therapy for primary aldosteronism can resolve the excess cardiovascular risk. In our patient, the reduction of plasma aldosterone level may significantly lower his increased cardiovascular risks.

## Conclusions

A high clinical index of suspicion for primary aldosteronism despite negative initial screening plasma aldosterone/renin ratio results and equivocal saline infusion test should encourage clinicians to pursue further investigations to establish the diagnosis of primary aldosteronism. Early recognition of the condition and optimal treatment of the mineralocorticoid excess can potentially cure or improve aldosteronism-associated hypertension and rectify the increased risk of cardiovascular disease and events.
